# Perceived professional competence of clinical research coordinators

**DOI:** 10.1017/cts.2020.558

**Published:** 2020-11-16

**Authors:** Jay W. Rojewski, Ikseon Choi, Janette R. Hill, Se Jung Kwon, Jasmine Choi, Eunice Kim, Linda McCauley

**Affiliations:** 1 Department of Career and Information Studies, University of Georgia, Athens GA, USA; 2 Office of the Dean, Nell Hodgson Woodruff School of Nursing, Emory University, Atlanta GA, USA

**Keywords:** Professional competence, Clinical Research Coordinators, self-assessment, career navigation, alluvial diagram

## Abstract

**Introduction:**

This study examined the perceived competence of Clinical Research Coordinators (CRCs) using several conceptual frameworks. Accurate self-assessment of one’s professional competence is a critical component in the career navigation process and contributes to (a) identifying and securing professional development (training), (b) leveraging professional strengths, and (c) integrating self-knowledge into a comprehensive career plan.

**Method:**

A survey design gathered responses from a sample of 119 CRCs in a southeastern region of the USA Two conceptual frameworks were used to represent aspects of CRC professional competence: the eight Joint Task Force (JTF) competence domains, and perceptions of strengths and training needs from a list of 12 task categories.

**Results:**

The JTF domain with the lowest competence level was Development and Regulations, while the highest was Communication. Perceived competence increased incrementally with years of experience. Top strengths involved direct patient interaction and data management. Tasks in need of training included project management and reporting issues. Variations in responses were based on years of experience as a CRC.

**Conclusion:**

Our results demonstrate an association between the self-reported strengths and training needs of CRCs and experience. This information can contribute to the self-directed career navigation of CRCs.

## Perceived Professional Competence of Clinical Research Coordinators

The work of Clinical Research Coordinators (CRCs) can be quite demanding and requires multiple skill sets that include direct patient care and interaction, administration of research protocols, data management, and serving as a primary liaison between various stakeholders in clinical trials [1]. Recent literature has begun to inform our understanding of CRCs, but relatively limited information is available on how these professionals view their preparation, professional development, and available career pathways. While our awareness of CRC’s professional needs has begun to coalesce [1, 2], an additional inquiry is needed to better understand and further develop the professional competence and career navigation of CRCs. Therefore, the purpose of this study was to examine the perceived competence of CRCs concerning their major professional strengths and areas where additional training is needed. This information can be helpful in not only determining CRC’s immediate professional training needs, but can also inform career self-management and decision-making.

The Joint Task Force (JTF) for Clinical Trial Competency [3, 4] identified eight core domains of competence required for all clinical research professionals. These competence domains represent consensus efforts on a comprehensive set of broad categories of knowledge, skills, and attitudes considered essential in conducting clinical research, including science concepts and research design, ethics and participant safety, medicine development and regulations, clinical trials operations, study and site management, data management and informatics, leadership and professionalism, and communication teamwork. A great deal of effort has been invested in defining these competence domains, and they provide structure for addressing the preparation, professional development, and assessment needs of CRCs, a vital component of the research workforce. However, the scope of CRCs’ work demands is broad and varied, ranging from supporting roles with limited responsibilities to roles that reflect a broad range of responsibilities in the completion of a large proportion of the clinical trials process. The breadth of the CRC workforce has led to complexity in identifying and applying the JTF competence domains presenting significant challenges (see [5–7] for detailed descriptions).

Challenges related to the competence domains are compounded by the rapid growth of technology occurring in all spheres of our personal and professional lives. In fact, this phenomenon is changing the very nature of work itself [8–10]. The impact of technological growth on clinical trials can be seen in the plethora of new drugs, devices, and behavioral interventions that are being presented to the research community for review [11]. The ways that clinical trials are conducted are also being affected; for example, the emergence of *precision medicine* has begun to replace traditional randomized–double-blind–placebo–control group designs [12] and new technology is being developed to enhance the collection and management of data. These technological advances require constant updating of skills so that CRCs can successfully integrate new technology into clinical research. Undoubtedly, these changes not only complicate the ability of CRCs to execute their daily job duties, but also places stress on their ability to successfully maintain or advance their career.

Rapid changes in the USA research enterprise and subsequent changes to work throughout society place additional challenges on CRCs to identify and secure necessary professional preparation and ongoing training [11]. While professions in other health-related fields (e.g., physicians, pharmacists, nurses) rely on well-articulated, academic, and structured pathways for career preparation, professional development, and advancement, the general career path for CRCs is less well developed. In fact, the specific career path for a CRC is often dependent on location or investigator needs rather than guided by standardized professional expectations or experiences for preparation and advancement [2].

It is increasingly important that CRCs accurately assess their professional competence in order to (a) monitor and enhance their professional abilities and (b) navigate (i.e., plan, prepare for, and pursue) their career paths. The role of assessing and developing one’s own professional competence is recognized as an important component in a comprehensive approach to both the initial preparation and ongoing development of health professionals [12–14]. Eva and Regehr [15] asserted that we should not consider self-assessment as merely a judgment of competence, but rather as an important influence on self-regulation and the ability to reflect on one’s professional practice.

To support the professional development, training, and career navigation of CRCs, a better understanding is needed of their views about the profession, paths taken to enter the field and, later on, navigation of their career as a CRC. Since one factor in making informed career-related decisions is the ability to accurately assess professional competence, we examined the self-assessment of CRCs about their professional competence.

## Method

### Participants

A web-based survey was distributed to individuals with the job title or job duties associated with CRC working in one of the four medical and/or educational institutions in a southeastern USA metropolitan area. The survey was distributed via email or listserv to all 411 CRCs employed by the four institutions at the time of the survey. This group constituted our sampling frame. All study protocols were reviewed and approved by institutional IRB. The available data pool included personnel with various job titles, reflecting institutional differences, e.g., Clinical Research Practitioner, Clinical Research Nurse, and Clinical Research Assistant in addition to CRC. Despite different titles, all participants reported performing duties associated with the role of CRC.

The survey, constructed through Qualtrics [16] survey development software, was made available to eligible CRCs for 45 days from the time of the first contact. Each participant received up to three reminder emails. CRCs were asked to complete a questionnaire that requested information about personal background, and perceived professional competency using two frameworks: (a) the eight professional core competency domains developed by the JTF for Clinical Trial Competency [3, 4] and (b) 12 CRC task categories reflecting professional practice.

Results were examined for the sample as a whole and by years of experience. This more focused examination was deemed appropriate since experience is likely to influence the perceptions of CRCs about their competence (viz., strengths and training needs) because of the expertise that comes from practice and the repetition of tasks. Further, job duties usually change over time and with increased expertise, as well as a likelihood that job responsibilities increase with growing experience and competence.

### Data Collection

#### Perceptions of Competence: JTF Domains

Perceptions of professional competence were measured by asking CRCs to rate their perceived level of competence on each of the eight core competency domains developed by the JTF for Clinical Trial Competency [3, 4]. The list of competency domains acknowledged and incorporated a number of previously designed competency lists in an effort to identify a comprehensive set of broad categories of knowledge, skills, and attitudes considered essential to function within the field of clinical research. The domains included science concepts and research design, ethics and participant safety, medicine development and regulations, clinical trial operations, study and site management, data management and informatics, leadership and professionalism, communication, and teamwork.

For each domain, respondents indicated their current level of competence using a 5-point Likert-type scale developed by the National Institutes of Health [17]: 1 = *Fundamental awareness*: Possesses some knowledge of basic techniques and concepts required of CRCs; 2 = *Novice*: Possesses limited experience (acquired either in classrooms or on-the-job scenarios), but requires help when performing tasks in domain: 3 = *Intermediate* (Practical application): Able to successfully complete tasks in domain as requested. Help from experts may be required occasionally, but skills can usually be performed independently. 4 = *Advanced* (Applied theory): Able to perform tasks associated with domain without assistance. Recognized within the immediate organization as “a person to ask” when difficult questions arise in this domain; and, 5 = *Expert*: A recognized authority on skills/tasks in this domain. Routinely provides guidance, troubleshoots, and answer questions related to this area of expertise. A *Not applicable* response was also available for respondents who were not required to apply or demonstrate any particular competency. Cumulative competence scores ranged from 8 to 40 with higher total scores indicating a generally greater level of overall competence.

#### Perception of Competence: CRC Tasks Categories

Respondents were also asked to assess their levels of competence on 12 categories that reflected major tasks completed by CRCs (and representative of JTF competence domains). Task categories included patient recruitment and screening, patient logistical support during the study, collecting and managing study sample, tests and interventions, administration of medications, providing education, nursing activities, database (access, entry, manipulation), communication, writing, compliance, and supervision. These task categories were generated from a synthesis of open-ended responses to questions about critical CRC job duties obtained from a series of formative interviews with CRCs employed in a large clinical and translational research center. Task categories did not alter or override the JTF competence domain structure and were included to provide illustrations of specific work activities associated with the work of CRCs. For example, several task categories (writing, supervision, compliance) reflected elements of the JTF competence domain, *clinical study operations.* Three additional task categories (patient recruitment and screening, patient logistical support, and collecting/managing study samples) represented elements of the broad JTF competence domain, *study and site management.*

Respondents were asked to select three tasks to represent their top professional strengths and three tasks to indicate areas where additional training was needed. These responses were analyzed using descriptive data and the construction of alluvial diagrams to illustrate the relationships among respondents’ sets of perceived strengths and training needs, respectively. Alluvial diagrams depict relationships between categories (e.g., perceptions) and can indicate the flow of data through different paths [20]. In our case, alluvial diagrams visually depicted CRCs’ strength and training need response patterns. Data were analyzed for the entire sample and with four levels of experience.

## Results

Respondents included 119 CRCs (29.0% response rate) composed primarily of women (*n* = 100, 84.0%). Approximately half of the sample reported their race/ethnicity as White (*n* = 65, 54.6%), with another 21.0% (*n* = 25) identifying as African American. CRCs in the sample reported a mean age of 36.11 years (*SD* = 10.8 years), and a mean of 5.91 years (*SD* = 4.9 years) of experience as a CRC. For analysis, respondents were arranged into four categories based on their years of CRC experience: less than 3 years, *n* = 36 (30.3%); 3–5 years, *n* = 34 (28.6%); 6–10 years, *n* = 28 (23.5%); and 11 years or more, *n* = 21 (17.7%).

### Perceived Competence on JTF Competence Domains

Respondents assessed their professional competence on the JTF competence domains that encompass CRC job performance [3, 4]. The 5-point Likert-type response scale designed by NIH [17] was used to denote perceived competence. Table 1 provides descriptive data for the entire sample and the four experience categories.

**Table 1.** Clinical Research Coordinator (CRC) perceptions of competence using the Joint Task Force (JTF) core domains of clinical trial competency

Experience as CRC	Scientific concepts		Ethics/safety		Development/regulation		Clinical operations		Management		Data/informatics		Leadership/professionalism		Communication		Overall	
	*M*	*SD*	*M*	*SD*	*M*	*SD*	*M*	*SD*	*M*	*SD*	*M*	*SD*	*M*	*SD*	*M*	*SD*	*M*	*SD*
<3 years	2.78	1.02	3.56	0.91	2.11	1.03	3.11	1.01	2.33	1.71	3.19	1.10	3.25	1.11	3.72	1.03	3.01	0.57
3–5 years	3.21	0.59	3.77	0.82	2.62	0.96	3.85	0.86	3.21	0.95	3.88	0.88	3.62	0.85	3.91	0.97	3.51	0.46
6–10 years	3.29	1.15	3.93	0.72	2.79	1.26	4.04	0.84	3.82	1.25	3.86	1.01	4.04	0.74	4.21	0.74	3.75	0.47
>10 years	3.62	1.07	4.29	0.78	3.52	1.03	4.38	0.59	4.33	0.73	4.05	0.97	4.33	0.73	4.43	0.75	4.12	0.36
Overall	3.17	0.99	3.83	0.95	2.66	1.16	3.77	0.97	3.29	1.28	3.70	1.04	3.73	0.97	4.02	0.93	3.52	0.45

Response set (1–5, 1 = Fundamental awareness: Some knowledge of basic techniques and concepts; 2 = Novice: Limited experience, requires help; 3 = Intermediate (practical application): Completes tasks independently, only requires occasionally help; 4 = Advanced (applied theory): Performs tasks without assistance; and, 5 = Expert: Recognized authority).

A mean of 3.52 was calculated for the entire sample across the JTF domains indicating that, as a group, CRCs reported intermediate levels of competence (i.e., ability to perform work tasks independently with only occasional support). Overall scores for each JTF competence domain ranged from a low of 2.66 (*SD* = 1.2) for Development and Regulations to a high of 4.02 (*SD* = 0.9) for Communication.

As expected, perceptions of competence increased incrementally with years of experience. CRCs with the least experience (<3 years) reported the lowest overall competence (*M* = 3.01) compared to CRCs with 3–5 years (*M* = 3.51) or 6–10 years of experience (*M* = 3.75). The most experienced respondents reported the highest perceived competence. Their overall mean of 4.12 reflected advanced ability. The variability in competence assessments also decreased with additional experience, suggesting that CRCs’ perceptions of their professional competence began to coalesce over time.

### Perceived Competence on 12 Task Categories

Respondents assessed their top 3 professional strengths and top 3 training needs from a list of 12 task categories. The task categories were similar to the JTF competence domains [3, 4], but reflected traditional skill- or task-orientations more familiar to CRCs in the course of completing their professional duties.

Collectively, CRCs’ greatest strengths were (a) *patient recruitment and screening*, (b) *database management, and* (c) *communication* (see Table 2). While reasons for selecting specific tasks were not part of our data collection protocol, we speculate that these three tasks represent fundamental aspects of CRC job duties, regardless of level or expertise. In contrast, few CRCs reported (a) *administration of medication* or (b) *writing* as a professional strength.

**Table 2.** Clinical Research Coordinator (CRC) perceptions of strengths and training needs on 12 task categories^a^

CRC job tasks	Perceived strengths					Perceived training needs				
	<3 years^b^	3–5 years	6–10 years	11+ years	Totals	<3 years	3–5 years	6–10 years	11+ years	Total
Patient recruitment/screening										
*n*	23	18	15	9	65	5	3	2	2	12
*%*	63.9	52.9	53.9	42.9	54.6	13.9	8.8	7.1	9.5	10.1
Patient logistical support										
*n*	10	7	9	5	31	7	7	6	2	22
*%*	27.8	20.6	32.1	23.6	26.1	19.4	20.6	21.4	9.5	18.5
Collecting/managing samples										
*n*	10	10	9	4	33	8	9	4	7	28
*%*	27.8	29.4	32.1	19.0	27.7	22.2	26.5	14.3	33.3	23.5
Tests and interventions										
*n*	5	4	4	1	14	16	18	9	8	51
*%*	13.9	11.8	14.3	4.8	11.8	44.4	52.9	32.1	38.1	42.9
Administration of medications										
*n*	––	1	2	––	3	10	9	15	2	36
*%*		2.9	7.1		2.5	27.8	26.5	53.6	9.5	30.3
Providing education										
*n*	8	7	3	7	25	9	6	5	7	27
*%*	22.2	20.6	10.7	33.3	21.0	25.0	17.6	17.9	33.3	22.7
Nursing activities										
*n*	2	2	2	1	7	1	2	3	5	11
*%*	5.6	5.9	7.1	4.8	5.9	2.8	5.9	10.7	23.8	9.2
Database management										
*n*	20	21	11	11	63	3	3	3	2	11
*%*	55.6	61.8	39.3	52.4	52.9	8.3	8.8	10.7	9.5	9.2
Communication										
*n*	18	14	11	9	52	–	2	3	3	8
*%*	50.0	41.2	39.3	42.9	43.7		5.9	10.7	14.3	6.7
Writing										
*n*	2	3	6	2	13	20	16	15	14	65
*%*	5.6	8.8	21.4	9.5	10.9	55.6	47.1	53.6	66.7	54.6
Compliance										
*n*	8	8	9	11	36	6	11	6	2	25
*%*	22.2	23.5	32.1	52.4	30.3	16.7	32.4	21.4	9.5	31.0
Supervision										
*n*	2	7	3	3	15	23	16	13	9	61
*%*	5.6	20.6	10.7	14.3	12.6	63.9	47.1	46.4	42.9	51.3

a Respondents (*n* = 119) identified their top 3 strengths and top 3 training needs from a list of 12 job task categories.

b Column subtotals represent the proportion of respondents who identified that task as a strength or training need (number of responses divided by group/sample size).

Tasks with the greatest need for additional training included (a) *writing*, (b) *supervision*, and (c) *administration/management of tests and interventions*. While it is possible that respondents considered these tasks as areas of professional weakness, it is equally plausible that these tasks begin to assume greater importance as CRCs gain experience and take on greater responsibilities for managing clinical trials, supervising staff, and generating reports. The fluid nature of medical research and the introduction of new or modified clinical trial protocols will also require ongoing training in these three areas. Two categories, (a) *database management* and (b) *communication*, were the least cited tasks in need of additional training, presumably because these tasks are fundamental CRC job duties.

The *nursing activities* task category was considered a professional strength (5.9%) or area in need of training (9.2%) by relatively few CRCs, as a large segment of this workforce does not have nursing credentials. However, approximately one-quarter (23.8%) of the most experienced CRCs identified this category as an area requiring additional training. This situation may represent customary changes in job expectations for more experienced CRCs that include a greater emphasis on nursing-related activities.

**Fig. 1.**
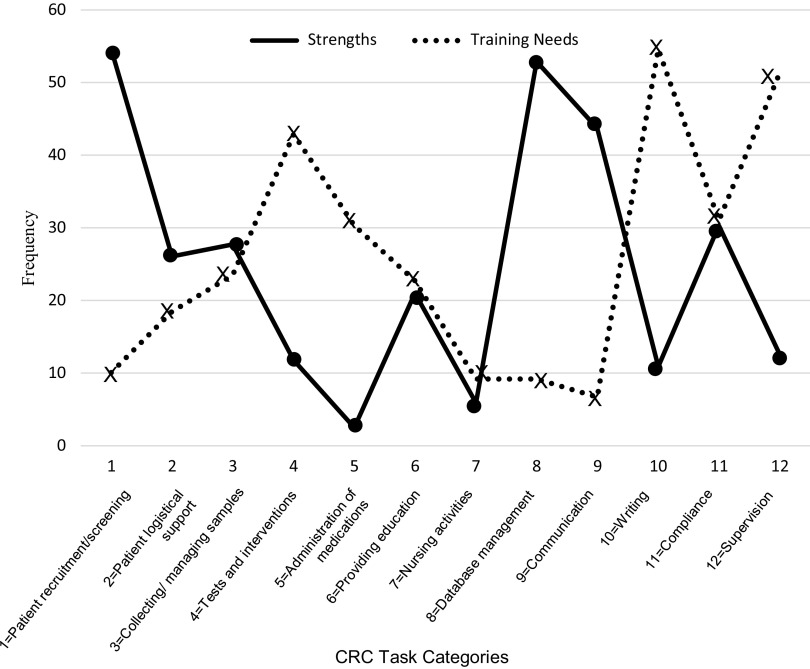
Response patterns of Clinical Research Coordinators (CRCs) for top three task strengths and top three tasks in need of training.

The frequency of CRCs’ perceived strengths and training needs were graphed to provide a visual representation of responses (see Fig. 1), revealing a reciprocal pattern for most strengths and training needs. The three tasks representing CRCs’ greatest strengths were also reported to be areas with low needs for additional training. Similarly, tasks where CRCs indicated a need for additional training were accompanied by lower levels of perceived strengths. While not direct evidence of valid self-assessment, this pattern seems reasonable and contributes to having confidence in CRCs’ ability to self-assess professional strengths and areas in need of improvement.

**Fig. 2.**
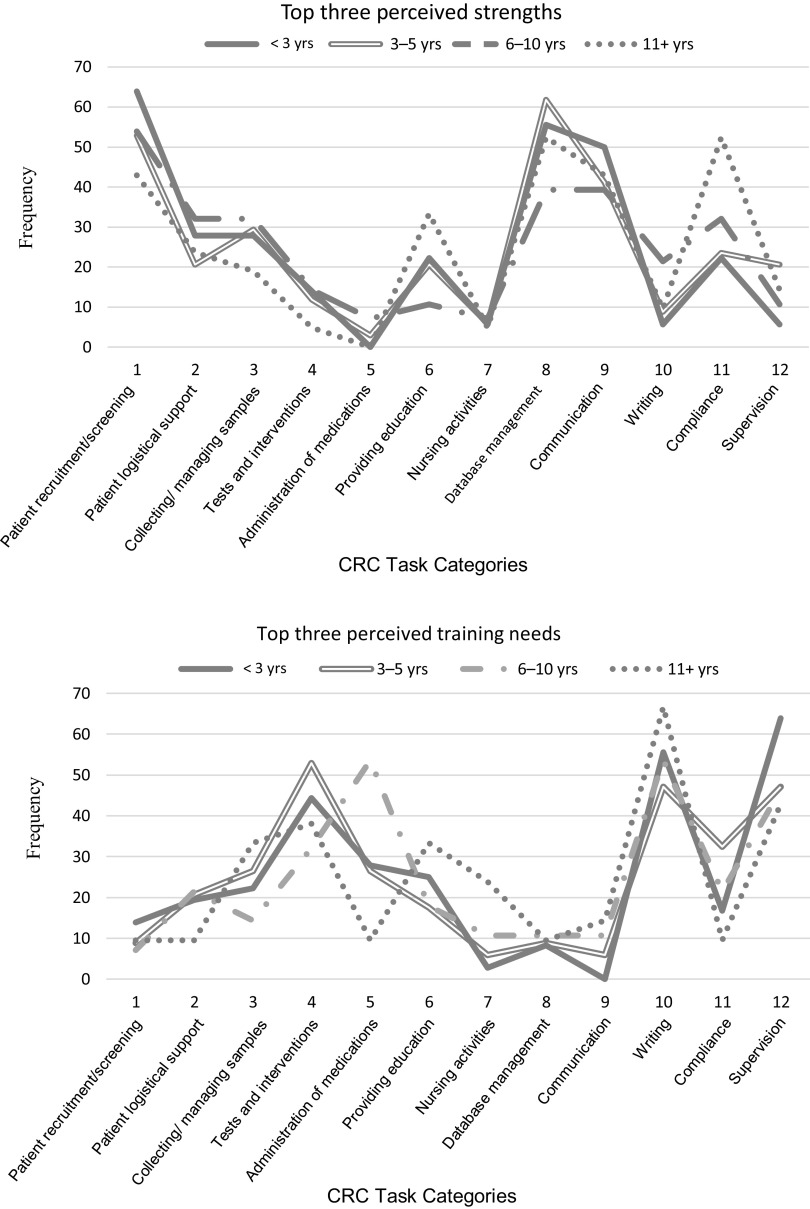
Clinical Research Coordinator (CRC) response profiles indicating the top 3 perceived strengths and top 3 perceived training needs among 12 task categories. *n* = 119 (responses = 357).

To examine how years of experience as a CRC might influence perceptions of strengths and training needs, the frequencies of responses by experience were graphed. The largest differences in perceived strengths were observed between CRCs with either the least or most experience. A larger number of CRCs with the least experience reported their professional strengths on tasks involving direct interaction with patients than their counterparts with the most experience. Conversely, experienced CRCs reported strengths on categories that represented administrative or reporting tasks. While unconfirmed, it is likely that these patterns reflect the types of tasks each group encounters in their daily work routines. Therefore, it is inappropriate to conclude that fewer experienced CRCs consider themselves competent on patient-related activities (or that fewer inexperienced CRCs consider themselves competent on administrative duties). An inverse pattern was found on the graph depicting areas of training need (see Fig. 2). The most experienced CRCs needed additional training in communication (including education) and writing. In contrast, the least experienced CRCs held a higher need for training on patient-related and supervisory tasks.

### Patterns of Responses on Perceived Competence

**Fig. 3.**
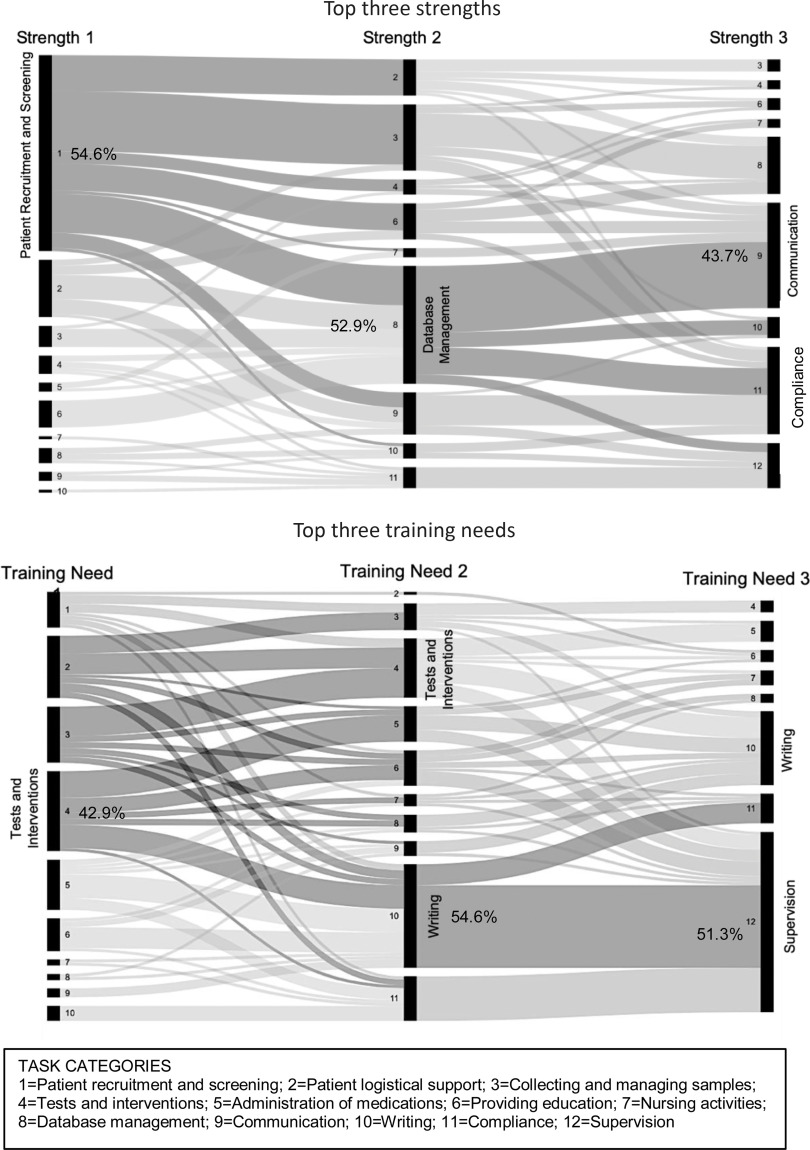
Profiles of the top 3 strengths and top 3 training needs of Clinical Research Coordinators (CRCs) from 12 task categories (entire sample).

Since CRCs were asked to indicate their top three professional strengths and top three areas in need of training (rather than rating competence on each task category), we examined the set or pattern of three strengths and three training needs, respectively. Alluvial charts provided a useful way to identify these patterns. We first looked at the sample as a whole. Fig. 3 contains alluvial diagrams that illustrate the frequency of responses indicating CRCs’ top three perceived strengths and top three training needs, respectively (Table 2 presents this data graphically). Each diagram is composed of 3 vertical steps representing each participant’s top 3 responses. Each of these steps contains up to 12 nodes representing the 12 task categories (task numbers do not imply ordinal value). The size of each node is proportional to the frequency of responses from CRCs. Each alluvial diagram depicts the collective flow or connections of CRC responses across the three task selections and also reflects frequencies associated with each task selection.

The top three professional strengths of CRCs were tasks that primarily involved direct patient interaction and data management. Roughly one-half of CRCs identified *patient recruitment and screening* (54.6%)*, database management* (52.9%), and *communication* (43.7%) among their top professional strengths. Other, less frequently identified task strengths included *patient logistical support*, *collecting–managing samples*, and *compliance*. The pattern of responses across these tasks reflects a fairly dominant pattern that incorporates some combination of these identified tasks (see Fig. 3).

Tasks identified as areas in need of additional training underscored project management and reporting issues. Approximately, one-half of CRCs identified one or more of their top three training needs in the areas of *testing and interventions* (42.9%), *writing* (54.6%), and *supervision* (51.3%). Other training needs, identified by approximately one-quarter of CRCs, included *collecting–managing samples*, *administration of medicines*, and *compliance.* The overall patterns of tasks needing additional training also reflected strong agreement on several tasks although some variety was also noted in Task 1 (see Fig. 3).

#### Perceived strengths by experience level

**Fig. 4.**
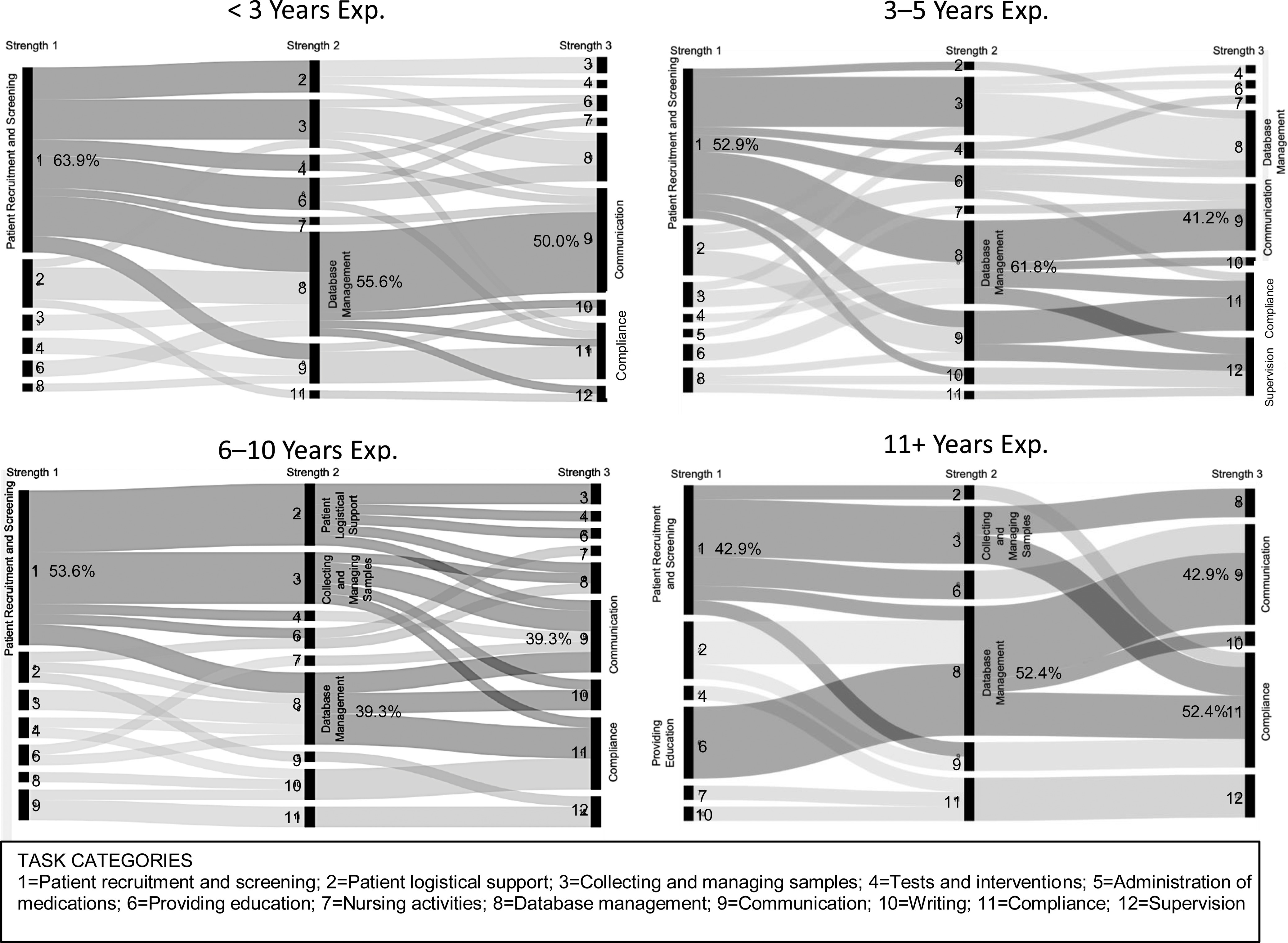
Profiles of the top 3 strengths of Clinical Research Coordinators (CRCs) from 12 task categories by years of experience.

Alluvial diagrams were used to examine the patterns of response for CRCs on perceived strengths based on professional experience. Patterns of expressed competence for CRCs with less experience (<3 years and 3–5 years) were similar to the pattern established for the entire sample. CRCs with greater levels of experience also reflected the predominant strengths pattern of the entire sample with several distinctions. First, CRCs with 6–10 years of experience reported greater variation in their top strengths compared to other groups. Second, the most experienced CRCs presented several clear patterns of response that reflected the overall sample but showed subtle differences (see Fig. 4).

#### Perceived training needs by experience level

**Fig. 5.**
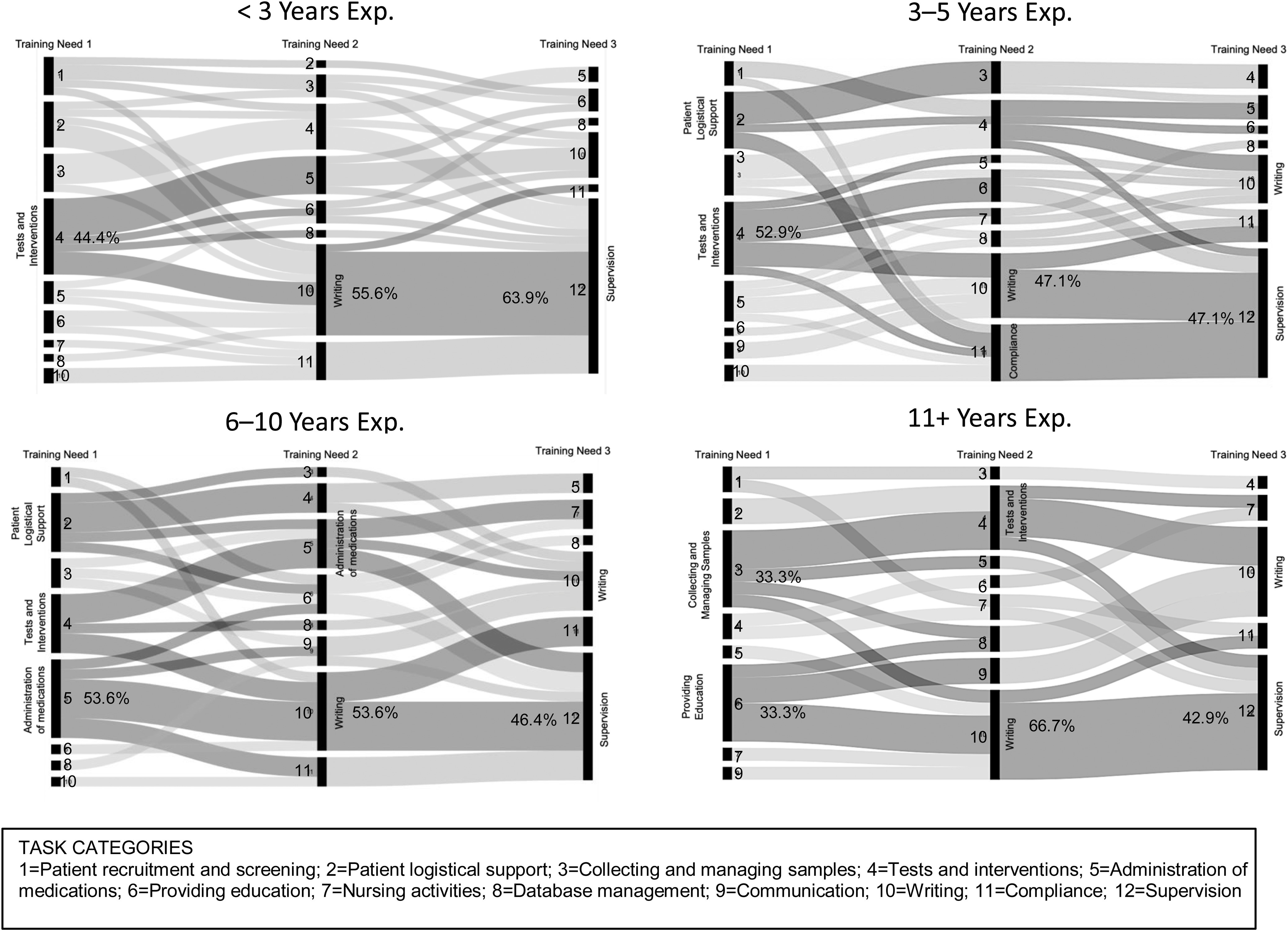
Profiles of the top 3 training needs of Clinical Research Coordinators (CRCs) from 12 task categories by years of experience.

Alluvial diagrams illustrate the patterns of response for CRCs’ perceived training needs based on professional experience. Patterns show that CRCs with the least experience (<3 years) presented a high degree of agreement about the top three tasks in need of training. Likewise, a high degree of agreement was observed for CRCs with the greatest levels of experience. CRCs with medium levels of experience showed much more variation in their patterns of training needs. Even so, the predominant patterns were likely to include one or more of the highest rated task categories (see Fig. 5).

## Discussion

Advances in medical technology and interventions are rapidly changing the ways that clinical trials are conceptualized, conducted, and reported. To stay current, CRCs must continually update their knowledge and skills through professional development and training. Demands for professional development are compounded by emerging shifts in the nature of work where responsibilities once assumed by institutions for managing and advancing employees’ career paths have shifted from organizations to individuals. As a result, CRCs (and others) are increasingly responsible for navigating their own career path (e.g., career-related planning, preparation, implementation, evaluation). Given this unfolding scenario, it is important that CRCs (a) accurately assess their current professional competence, (b) identify and secure available professional development opportunities for areas where additional training is needed, and (c) integrate these abilities into a broader career navigation strategy which they manage. Self-assessment of professional competence is an important component in the self-guided career navigation process. One step in understanding and supporting the self-assessment component of the career navigation process is to examine CRCs’ perceived professional competence.

Two frameworks were used for assessing CRCs’ professional competence. First, the eight domains of the JTF framework [3, 4] were used. As a group, CRCs reported an ability to independently apply knowledge and skills associated with each of the JTF domains, requiring only occasional help. Communication, ethics/safety, clinical operations, data/informatics, and leadership/ professionalism domains were among the highest rated areas of strength. CRCs with the least experience consistently rated their competence lower, while those with the most experience rated their competence higher than all other experience groups across all eight domains. While we did not specifically test the validity of perceptions, our results may be at odds with prior literature [17, 19] that identified potential concerns with the accuracy of self-assessing job performance. Although preliminary, the CRCs in our sample were able to assess their competence in ways consistent with professional experience (and expected competence) and could be used to guide professional development and career-related plans.

Several points may explain the present findings, as well as support future research efforts. First, the JTF competence framework [3, 4] represents broad categories of knowledge, skills, and attitudes considered essential for clinical research. The comprehensive nature of the framework provides structure and organization for understanding the breadth of CRC professional competence but may not provide the best platform for providing detailed assessments of competence needed for career planning. Second, the NIH-based rating scale [17] provided an externally valid and easily understood standard for assessing professional competence. However, the response options may have been too imprecise to make finer distinctions between CRCs’ competence. While most respondents indicated a general ability to independently complete their job duties with only minimal supervision (as would be expected), responses did not provide additional detail or explanation of this assessment. For example, a CRC may be able to perform job duties independently but feel less confident on specific tasks or experience some hesitation in conducting specific aspects of the task. These more detailed descriptions would be useful for pinpointing necessary training or support.

The second framework employed to assess perceived competence asked respondents to identify top professional strengths and areas needing additional training from a list of 12 task categories. These categories were complementary to (and, in some cases overlapped with) the JTF competence domains. Tasks were used because CRCs are likely to be more familiar with traditional skill- or task-orientations when conceptualizing or executing job duties. The relationships between tasks identified as top strengths and the corresponding low levels of training need suggests that respondents were able to determine whether performance on a particular task category constituted an asset or required support. The ability to accurately gauge professional abilities (and areas requiring support or additional training) is critical when determining which professional development opportunities to pursue and planning for future career goals. CRCs in our study assessed current levels of competence. Next steps would be to determine if this self-knowledge can be applied to selecting necessary training for improved job performance or decision-making about future career goals.

Myriad combinations of CRCs’ top strengths and training needs were generated. However, broad profiles emerged in our analysis. For a majority of CRCs, the profile of top strengths included one or more of the four task categories: patient recruitment and screening, database management, communication, and compliance. Similarly, the training needs profile for a majority of respondents included one or more of three specific tasks: tests and interventions, writing, and supervision. Information regarding training needs can be especially helpful in focusing on institutional training efforts, as well as enhance the career decision-making of CRCs.

Years of experience not only contributes to a maturing sense of competence but is likely to be accompanied by an evolution of CRCs’ job responsibilities. To determine how the experience influenced CRCs’ perceived competence, task profiles were constructed for four groups based on years of experience. Alluvial diagrams offered an opportunity to gain a sense of the flow and connectedness (i.e., profiles) of CRCs’ responses to top strengths and training needs. Several trends were observed.

The strengths profile for the least experienced CRCs was more well-defined than other groups. More than half of respondents with less than 3 years of experience identified patient recruitment and screening, database management, and/or communication as top strengths. The profiles of CRCs with 3–5 years of experience and those with 6–10 years of experience were similar (top strengths for both groups included patient recruitment and screening, communication, and compliance). However, two-thirds of CRCs with 3–5 years of experience reported database management as a strength while less than half of the more experienced CRC group identified this task. The strength profile of the CRC group with 6–10 years of experience varied to a greater extent than the less experienced group suggesting a wider range of job responsibilities. The strengths profile of CRCs with the most experience was more focused than the patterns of CRCs with 3–10 years of experience.

The profile patterns of training needs for CRCs with the least experience were rather clear and consistent; testing interventions, writing, and supervision were the predominant components of most CRCs in this group. The patterns of CRCs in the other three experience groups showed less uniformity but several training needs were consistent in all profiles, including testing interventions, writing, and supervision. These tasks, in particular, assume a greater portion of CRCs’ job duties as they gain experience and responsibilities for managing clinical trials. Additional information about the connections between specific job duties and reported competence is warranted.

Despite persistent calls for including self-assessment in professional preparation and development, the literature advocates caution when considering the validity of self-assessment results. Sitzmann et al. [18] reported relatively weak correlations between self-assessments of cognitive outcomes and actual performance, while somewhat stronger correlations were reported for the self-assessment of affective outcomes, particularly satisfaction and motivation. Interestingly, the validity or accuracy of self-assessment can be influenced by the respondents’ level of performance, i.e., the *Dunning–Kruger effect* [19]. Poorer performing individuals are more likely to overestimate their competence, while higher performing individuals tend to underestimate competence levels. In general, the validity of self-assessments vary depending on specific content, contexts, and perspectives [15]. Regardless of concerns about validity and reliability, the use of self-assessment remains an important component in professional development and provides a mechanism to better understand how CRCs view their professional ability. These perceptions are likely to influence decisions about pursuing particular professional development opportunities and contribute to determining career paths.

While the present study contributes to a better understanding of how CRCs perceive their professional competence, several limitations are noted. Our sample was not selected at random and did not represent a large-scale population (e.g., regional or national). The obligatory caution about self-report data is recognized (i.e., social desirability). Although results are suggestive that this issue may not be a prominent threat to interpretation, we did not validate CRCs’ assessments with objective (external) sources. Therefore, our findings do not suggest that individual assessments of professional competence be substituted for objective evaluations of professional competence or training needs. Future studies could address this issue by comparing personal assessments with objective (external) evaluations of professional competence. Future studies might also consider incorporating information about the past professional experience (e.g., types of clinical trials) and disciplines of CRCs to determine the possible associations between these factors and perceived professional competence.

While the generalizability of results beyond the current sample is not warranted, current findings do inform us about CRCs’ perceptions of the competence using the established competence framework. Particularly, areas of perceived training needs might support the provision of necessary professional development in a timely and more efficient manner. Additional research to extend the present results to a national sample of CRCs is needed.

## References

[1] Scala E , Price C , Day J. An integrative review of engaging clinical nurses in nursing research. Journal of Nursing Scholarship 2016; 48: 423–430. 27232935 10.1111/jnu.12223

[2] Speicher LA , et al. The critical need for academic health centers to assess the training, support, and career development requirements of clinical research coordinators: Recommendations from the Clinical and Translational Science Award Research Coordinator Taskforce. Clinical and Translational Science 2012; 5: 470–475. 23253669 10.1111/j.1752-8062.2012.00423.x PMC3531899

[3] Joint Task Force for Clinical Trial Competency. *Harmonized Core Competency Framework [Ver. 2.0]*, [Internet] 2017. (http://mrctcenter.org/wp-content/uploads/2017/12/2017–12–07-Core-Competencies-V2.0–8-Summary.pdf)

[4] Sonstein SA , et al. Moving from compliance to competency: A harmonized core competency framework for the clinical research professional. Journal of Clinical Research Best Practices 2014; 10: 1–12.

[5] Gwede CK , Johnson DJ , Trotti A. Measuring the workload of clinical research coordinators, Part 1: Tools to study workload issues. Applied Clinical Trials 2000a; 9: 40–44.

[6] Gwede CK , Johnson DJ , Trotti A. Measuring the workload of clinical research coordinators, Part 2: Workload implications for sites. Applied Clinical Trials 2000b; 9: 42–47.

[7] Gwede CK , Johnson DJ , Roberts C. , Cantor AB. Burnout in clinical research coordinators in the United States. Oncology Nursing Forum 2005; 32: 1123–1130. 16270108 10.1188/05.onf.1123-1130

[8] Ross A. The Industries of the Future. New York: Simon and Schuster, 2016.

[9] Savickas M. The theory and practice of career construction. In: Brown SD, Lent RW, eds. Career Development and Counseling. Hoboken, NJ: Wiley and Sons, 2005, pp. 42–70.

[10] Susskind R , Susskind D. The Future of the Professions: How Technology will Transform the Work of Human Experts. New York: Oxford University Press, 2015.

[11] Kolb HR , Kuang H , Behar-Horenstein LS. Clinical research coordinators’ instructional preferences for competency content delivery. Journal of Clinical and Translational Science 2018; 2: 217–222. 30820358 10.1017/cts.2018.320 PMC6382289

[12] Hornung CA , et al. Competency indices to assess the knowledge, skills, and abilities of clinical research professionals. International Journal of Clinical Trials 2018; 5: 46–53.

[13] Lew MDN , Alwis WAM , Schmidt, HG. Accuracy of students’ self-assessment and their beliefs about its utility. Assessment and Evaluation in Higher Education 2010; 35: 135–156.

[14] Root Kustritz MV , Molgaard LK , Rendahl A. Comparison of student self-assessment with faculty assessment of clinical competence. Journal of Veterinary Medical Education 2011; 38: 163–170. 22023925 10.3138/jvme.38.2.163

[15] Eva KW , Regehr G. Self-assessment in the health professions: A reformulation and research agenda. Academic Medicine 2005; 80: 46–54. 10.1097/00001888-200510001-00015 16199457

[16] Qualtrics. *Online Survey Software* [Internet], 2018. (https://www.qualtrics.com/research-core/survey-software/)

[17] National Institutes of Health, Office of Human Resources. *Competency Proficiency Scale* [Internet], 2019 [cited Aug 1, 2020]. (https://hr.nih.gov/working-nih/competencies/competencies-proficiency-scale)

[18] Sitzmann T , Ely K , Brown KG , Bauer KN . Self-assessment of knowledge: A cognitive learning or affective measure? Academy of Management Learning and Education 2010; 9: 169–191.

[19] Ehrlinger J , Johnson K , Banner M , Dunning D , Kruger J. Why the unskilled are unaware: Further explorations of (absent) self-insight among the incompetent. Organizational Behavior and Human Decision Processes 2008; 105: 98–121. 19568317 10.1016/j.obhdp.2007.05.002 PMC2702783

[20] RAWGraphs Team. *How to Make an Alluvial Diagram* [Internet], 2020 [Licensed under CC BY-NC-SA 4.0]. (https://rawgraphs.io/learning/how-to-make-an-alluvial-diagram/)

